# 

**Published:** 1878-07

**Authors:** 


### CONTENTS.

### COMMUNICATIONS.

Oral Electricity. *By J. S. Cassidy, D. D. S.,* ..... 265
Pivot Teeth. *By E. Peabody,* ..... 271
Professional Duties of the Dentist. *By C. L. Kelsey, D. D. S.,* ..... 278
Transplanting. *By G. R. Thomas, D. D. S.,* ..... 281

### PROCEEDINGS OF SOCIETIES.

Discussions before the Kentucky State Dental Society ..... 298
Pennsylvania State Dental Society ..... 305

### EDITORIAL.

Alumni Reunion ..... 307
Resignation and Reorganization ..... 309
Obituary ..... 310
Ho, To American Dental Association ..... 310

